# Effects of occupational status on social adjustment after laryngectomy in patients with laryngeal and hypopharyngeal cancer

**DOI:** 10.1007/s00405-019-05378-9

**Published:** 2019-03-29

**Authors:** Kumiko Kotake, Ichiro Kai, Kazuyo Iwanaga, Yoshimi Suzukamo, Aya Takahashi

**Affiliations:** 1 Faculty of Nursing, Nara Medical University, 840 Shijou-cho, Kashihara, Nara Japan; 2 Graduate School of Medicine, Faculty of Medicine, The University of Tokyo, 7-3-1, Hongo, Bunkyo-ku, Tokyo, Japan; 3 School of Nursing, Faculty of Medicine, Fukuoka University, 8-19-1, Nanakuma, Jonan-ku, Fukuoka, Fukuoka Japan; 4 Physical Medicine and Rehabilitation, Tohoku University School of Medicine, 2-1-1 Seiryo-cho, Aoba-ku, Sendai, Miyagi Japan; 5 Department of Nursing, Saitama Prefectural University, 820, Sannomiya, Koshigaya, Saitama Japan

**Keywords:** Laryngectomized patients, Occupation, Social adjustment, Laryngeal and hypopharyngeal cancer, Quality of life

## Abstract

**Purpose:**

This study was performed to examine the relationship of social adjustment with occupation and life changes in patients with laryngeal and hypopharyngeal cancer, from before laryngectomy to 1 year after hospital discharge.

**Methods:**

The subjects were 27 patients with laryngeal and hypopharyngeal cancer who were admitted to hospital for laryngectomy and provided informed consent for participation in the study. The patients answered questionnaire surveys before surgery, and 3, 6, and 12 months after hospital discharge. Regarding social adjustment, social functioning (SF) and mental health (MH) in SF-36V2 were used as dependent variables, and time, occupation status, age, family structure, and sex as independent variables. Repeated measures analysis of variance was used to examine the main effect, and second- and third-order interactions were also examined.

**Results:**

The age of the subjects was 62.9 ± 6.4 years and about 30% had an occupation. Loss of voice was the reason for 30% leaving work. In an examination of the main effects of the four variables, only age was significant regarding SF, and SF was favorable in subjects aged ≥ 64 years old. Regarding MH, age and family structure were significant, and MH was higher in older subjects who lived alone. The interaction between time and the other 3 variables was not significant. Only time/age/occupation was significant for MH. Regarding SF, a weak interaction was suggested, but it was not significant.

**Conclusion:**

Older subjects showed better social adjustment, and those who lived alone had better MH. These findings may have been due to a reduced environmental influence. MH of subjects with an occupation decreased more at 3 months or later after hospital discharge, compared to those without an occupation. Especially for younger patients, development of new approaches is required to allow families and colleagues of patients to understand the difficulties of patients with laryngeal and hypopharyngeal cancer.

## Introduction

Laryngectomized patients with laryngeal and hypopharyngeal cancer face fear of a malignant tumor, anxiety regarding recurrence and complications after surgery, while also having physical, mental, and social difficulties due to communication disorder caused by sudden loss of voice after surgery [1–5]. Large mental burdens are caused by difficulty in communicating, and patients may give up maintaining relationships with others and even with family members because of their inability to talk [6, 7]. Laryngeal and pharyngeal cancer also causes problems with eating (dysphagia, dysgeusia, etc.), egesting, sleeping, and other basic activities, in addition to inability to speak with a real voice, and patients have anxiety and depression after hospital discharge [4, 8–11]. Laryngectomized patients face difficulties in daily life that they could not imagine before surgery and this reduces quality of life (QOL) through withdrawal after discharge [5, 12–15].

As one form of social rehabilitation, patients may return to work after laryngectomy, but it is likely that they will have considerable difficulties in working after hospital discharge. Furthermore, patients with no occupation may have problems related to motivation in life. For both, some support may be required and this support requires classification based on occupation status, if QOL differs depending on this status. However, there have been no studies of QOL related to occupation status in patients after laryngectomy. Therefore, in this study, we examined the effects of occupation on QOL using social functioning (SF) and mental health (MH) as indexes. In addition, the relationships of social adjustment with occupation and life changes from before surgery to 1 year after discharge have not been examined in these patients. Therefore, the objective of this study was to examine these relationships in patients treated with laryngectomy for laryngeal and hypopharyngeal cancer.

## Materials and methods

For the present analysis, we used the data of the patients who had completed all the four surveys (*N* = 27). The subjects were hospitalized patients with laryngeal and pharyngeal cancer who were scheduled to undergo laryngectomy. All subjects provided informed consent for participation in the study. A total of 27 subjects answered questionnaire surveys before surgery and 3, 6, and 12 months after discharge.

### Social adjustment

The surveys were performed in hospital before surgery, and by mail after discharge. Social adjustment was examined using social functioning (SF) and mental health (MH) in the SF-36V2 Japanese version, which has been standardized and widely used in Japan [16–18]. Regarding SF, the subjects were asked whether and how much their usual communications with family members, friends, neighbors, and other people were affected for physical or mental reasons. Regarding MH, the subjects were asked if they had been nervous and had been in a gloomy mood during the past month. When the responses for SF-36V2 items were missing, standardized-scoring algorithm was used for imputation of SF and MH, and the Norm-based Scoring values was calculated. Occupation status and reasons for leaving work were investigated as markers of social activity.

### Basic characteristics

Age, sex, and family structure were recorded as background factors. Diagnosis, stage of cancer, surgical details, lymphadenectomy, and physical conditions at discharge (trachea hole construction, perilaryngeal edema, constipation) were obtained from medical records. Repeated measures analysis of variance was used to analyze data, with SF and MH in SF-36V2 as dependent variables of social adjustment, and occupation status, age, sex, and family structure as independent variables reflecting social activity. In this analysis, social adjustment was calculated using a standardized scoring program, and norm-based scores (NBS) were used to examine social adjustment. The main effect of the independent variables was calculated and then interactions were examined. However, since the subjects included only 4 females, we did not examine an interaction with sex. Regarding family structure, the subjects were divided into two groups, those living alone and those living with one or more family members. For age, the subjects were divided into groups aged ≤ 63 and ≥ 64 years old based on the median age. The presence of lymph node dissection, disease stage, and therapeutic method could also have been evaluated as causal factors, but due to the distribution bias and the small number of subjects, only diagnosis was used as this type of factor. Thus, the subjects were divided into laryngeal cancer and hypopharyngeal cancer groups.

Significance was determined to be ≤ 5%. Data with a ≤ 10% probability were viewed as showing a tendency, but not as significant.

1. The main effect test examined time, age, occupation status, family structure (two groups), diagnosis (two groups), and sex using repeated-measures ANOVA.
2. Second-order interactions between time and four other variables: age, occupation status, diagnosis (two groups) and family structure (two groups).
3. Third-order interactions were examined among three variables: time/occupation/age, time/occupation/family structure, and time/occupation/diagnosis. Stratification based on occupation status was used to show the time course of social adjustment graphically. In the analysis, multiple comparisons were performed for items with a significant effect.

## Results

### Background of subjects, surgical details, and conditions at discharge

The subjects (23 males and 4 females, Table 1) were aged 62.9 ± 6.4 years (range 48–76 years) and included 16 subjects aged ≤ 63 years (59.3%). Regarding family structure, 22 subjects lived with one or more family members, and 5 lived alone. Six subjects (22.2%) were diagnosed with laryngeal cancer, including 2 (7.4%) with recurrent cancer, and 20 (74.1%) were diagnosed with hypopharyngeal cancer, with disease stages IV and III in 25 (92.6%) and 1 (3.7%) subjects, respectively. Total laryngectomy + esophageal reconstruction, preoperative radiotherapy + total laryngectomy, and postoperative radiotherapy + total laryngectomy were performed in 24 (88.9%), 1 (3.7%), and 1 (3.7%) subjects, respectively. Lymph node dissection was performed in 26 subjects (96.3%). Permanent tracheostoma, dysphagia, constipation, dysgeusia, anosmia, cervical skin problems, facial edema, and arm edema occurred in 5 (18.5%), 16 (59.3%), 11 (40.7%), 5 (18.5%), 4 (14.8%), 5 (18.5%), 5 (18.5%), and 4 (14.8%) subjects, respectively.

**Table 1:** Basic characteristics (*N* = 27)

	*N* (%)
Age [Mean ± SD (range): 62.9 ± 6.4 (48–76) ]	
≦ 63 years	16 (59.3)
≧ 64 years	11 (40.7)
Sex	
Male	23 (85.2)
Female	4 (14.8)
No. of family members	
Alone	5 (18.5)
2 ≧	22 (81.5)
Diagnosis	
Laryngeal cancer	6 (22.2)
Supraglottic cancer	4 (14.8)
Recurrence of cancer	2 (7.4)
Hypopharyngeal cancer	20 (74.1)
No response	1 (3.7)
Stages of Cancer	
Stage III	1 (3.7)
Stage IV	25 (92.6)
No response	1 (3.7)
Surgery	
Total laryngectomy with reconstruction of esophagus	24 (88.9)
Radiotherapy before total laryngectomy	1 (3.7)
Radiochemotherapy after total laryngectomy	1 (3.7)
No response	1 (3.7)
Lymphadenectomy	
With lymphadenectomy	26 (96.3)
No response	1 (3.7)

Among the 27 subjects, 9 (33.3%), 8 (29.6%), 9 (33.3%), and 7 (25.9%) had an occupation before surgery and at 3, 6, and 12 months after hospital discharge, respectively, with no significant difference among the time points (Table 2). Regarding the reason for leaving work, 5 subjects (27.8%) answered loss of voice that had occurred before surgery, and one subject each answered depression, restructuring, and disease. One year after discharge, 8 subjects (40%) had left their work due to loss of voice. The data in Table 2 show the total numbers of subjects in and out of work at each time point. These data show that a few subjects lost and then regained work over the study period. The reasons for leaving work were those given by the subjects when asked this question at each time point.

**Table 2:** Occupation status and reasons for leaving occupation (*N* = 27)

	Before surgery	3 months after discharge	6 months after discharge	1 year after discharge
Subjects with occupation	9 (33.3)	8 (29.6)	9 (33.3)	7 (25.9)
Subjects without occupation	18 (66.7)	19 (70.4)	18 (66.7)	20 (74.1)
Reason for leaving work^a^				
Retirement	11 (61.1)	13 (68.4)	11 (61.1)	12 (60.0)
Loss of voice	5 (27.8)	5 (26.3)	5 (27.8)	8 (40.0)
Depression	1 (5.6)	1 (5.3)	1 (5.6)	1 (5.0)
Restructuring	1 (5.6)	1 (5.3)	1 (5.6)	1 (5.0)
Disease	1 (5.6)	1 (5.3)	1 (5.6)	1 (5.0)

Data are shown as *N* (%)

^a^Multiple answers are included skin problems in 5 (18.5%), facial edema in 5 (18.5%), and upper extremity edema in 4 subjects (14.8%)

### Changes in social adjustment

An analysis of social adjustment in the period from before surgery to 1 year after hospital discharge (Table 3) showed that social functioning (SF) decreased significantly from 3 months after discharge and this continued until 1 year, whereas there were no major changes in mental health (MH).

**Table 3:** Social adjustment based on norm-based scores (*N* = 27)

	Before surgery	3 months after discharge	6 months after discharge	1 year after discharge
Social functioning (SF)	42.0 ± 13.3*	33.5 ± 12.8*	36.1 ± 14.2	39.3 ± 14.6
Mental health (MH)	47.3 ± 8.8	46.4 ± 12.0	46.5 ± 10.0	46.8 ± 12.4

Data are shown as mean ± S.D

*indicates *p* < 0.05

**Fig. 1:**
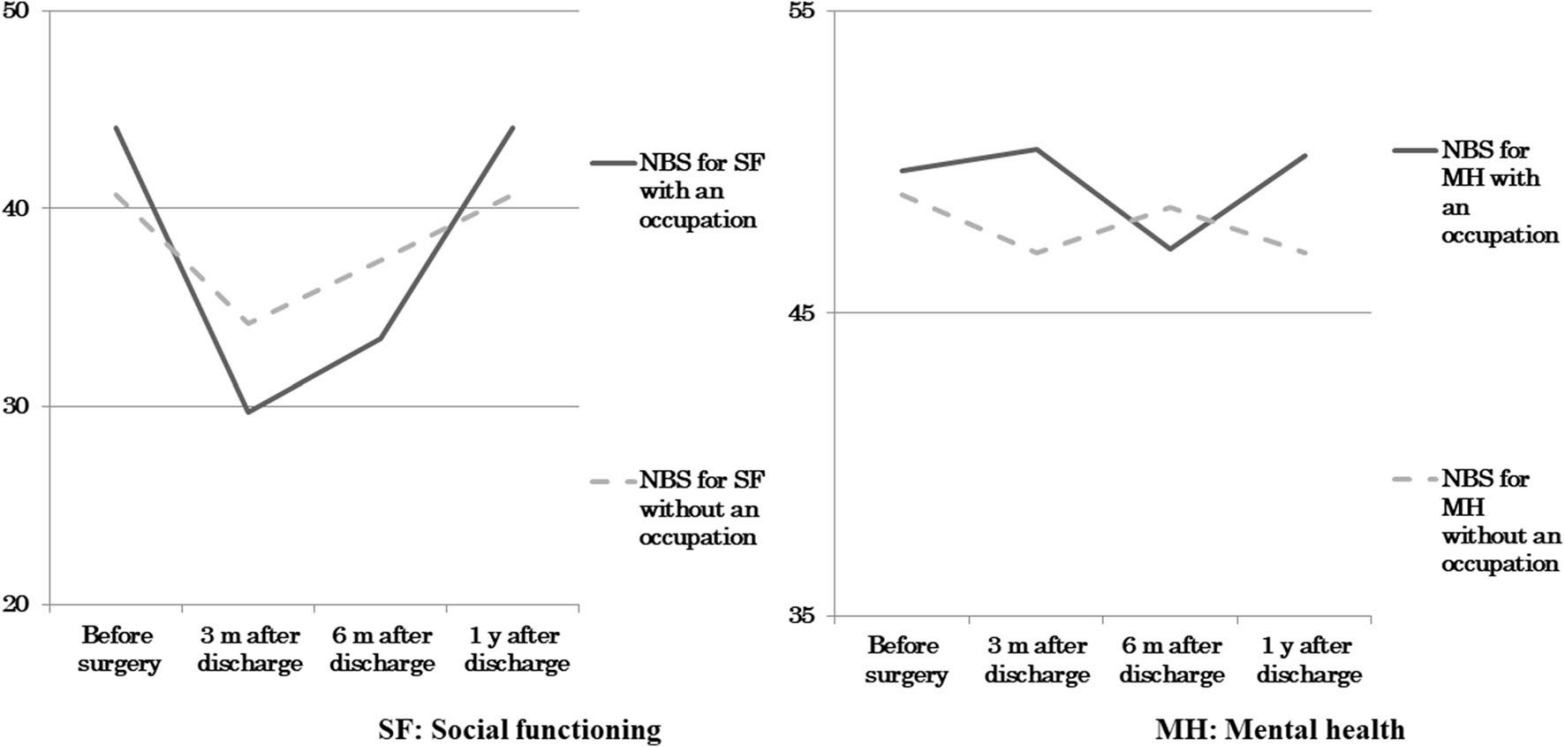
Time course of social adjustment in subjects with and without an occupation using norm-based scores (NBS: least mean square)

This general pattern of SF changes was similar in subjects with and without an occupation, but SF in those with an occupation showed a particularly significant decrease at 3 months after hospital discharge [least mean square (LMS) = 29.7 points], compared to those without an occupation (Fig. 1). There were no major changes in MH in subjects with and without an occupation. MH in subjects with an occupation decreased more than those without an occupation at 6 months after discharge, but increased 1 year after discharge.

#### Main effect test

A main effect for SF was confirmed for age (*p* = 0.0046), time (*p* = 0.0859), and diagnosis (*p* = 0.0787) (Table 4), while that for MH occurred for age (*p* < 0.0001) and family structure (*p* = 0.0014). In the main effect test for four variables (Table 4), a main effect for SF was found for age (*p* = 0.0046), with subjects aged ≥ 64 years old having more favorable SF. Since a small effect was found for time (*p* = 0.0859) and diagnosis (*p* = 0.0787), although it was not significant, multiple comparisons were performed.

**Table 4:** Main effects for social functioning and mental health

Model	Social functioning	Mental health
Time	☨	−
Occupation	−	−
Age	**	***
Family structure	−	***
Diagnosis	☨	−

^☨^*p* < 0.1, *p < 0.05, **p < 0.01, ***p < 0.001, – no significant difference

SF was more favorable in subjects aged 64 years or older. A small, but not significant, effect was found for time and diagnosis and, therefore, multiple comparisons were performed. This showed that the effect 3 months after hospital discharge (LMS: 33.5 points) was significantly lower than that before surgery (LMS: 42.0 points) (*p* < 0.05). SF in the hypopharyngeal cancer group (LMS: 37.3 points) was lower than that in the laryngeal cancer group (LMS: 42.8 points). In addition, MH of subjects who lived with one or more family members (LMS: 44.8 points) was lower than that in those who lived alone (LMS: 53.0 points). Thus, MH was better in older subjects who lived alone.

#### Second-order interactions

There was no second-order interaction between time and the other four variables.

#### Third-order interactions

**Fig. 2:**
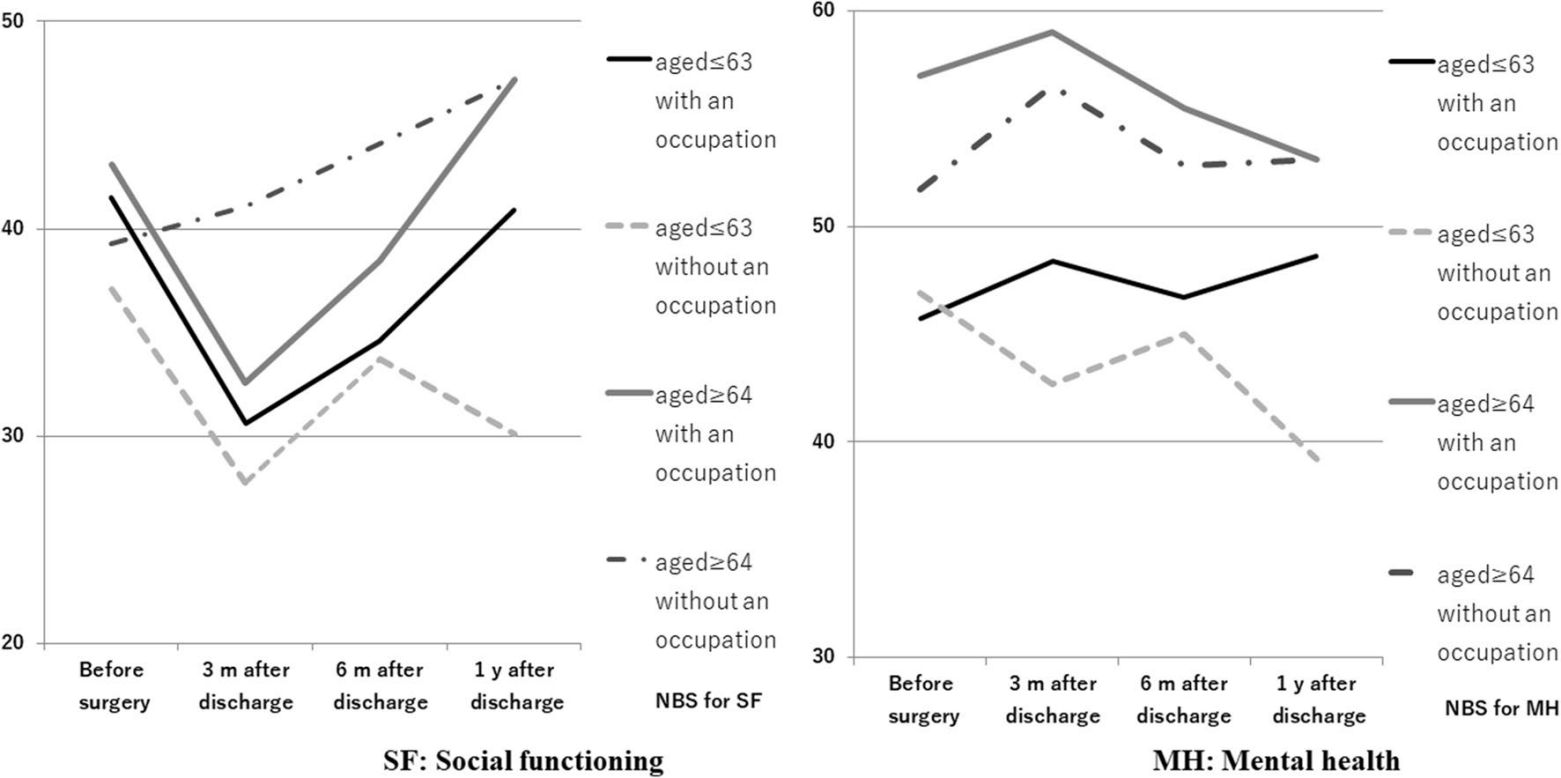
Age-based time course and interaction of social adjustment (SF and MH) in subjects with and without an occupation

Significant third-order interactions were found for time/age/occupation for SF (*p* = 0.0447) and MH (*p* = 0.0061). SF and MH were both higher in older subjects. Multiple comparison for time/occupation/family structure at different time points showed time-dependent changes for SF and MH after laryngectomy in subjects who had an occupation and lived with one or more family members, although these changes were not significant. The SF score at 3 months after hospital discharge (LMS 31.1) was lower than that before surgery (LMS 40.5), and the score at 6 months after discharge (LMS 34.2) was significantly lower than that before surgery. MH at 3 months after discharge in subjects with no occupation who lived with their family (LMS 43.2) was significantly lower than that for subjects who had an occupation and lived with their family (LMS 59.1). Figure 2 shows a graph of time-dependent changes in social adjustment (SF and MH) in the subjects divided into older and younger groups based on the mean age, and then subdivided based on occupation status.

SF in subjects with and without an occupation showed a significant decrease 3 months after discharge regardless of age, except for older subjects without an occupation. MH in subjects with and without an occupation showed no change even at 1 year after discharge, compared to data obtained before surgery, except for younger subjects without an occupation, who had an LMS of 39.2 at 1 year after discharge.

The younger subjects without an occupation had lower scores for both SF and MH than those in the other three categories, except for MH before surgery. Such subjects showed significantly lower SF at 3 months (LMS 27.8) and 1 year (LMS 30.1) after discharge, compared to an LMS of 30.6 for younger subjects with an occupation at 3 months after discharge.

SF in older subjects with an occupation was higher (LMS 43.1) before surgery compared to other subjects, but was lower (LMS 32.6) at 3 months after discharge. In contrast, older subjects without an occupation had scores in the 30-point range until 6 months after discharge, and these scores continued to increase beyond 3 months after discharge. MH in these subjects was also better than that in subjects in the other three categories. All older subjects lost their employment due to loss of voice and had no occupation at 1 year after discharge.

## Discussion

In this study, ≥ 74% of the 27 subjects had hypopharyngeal cancer and the disease stage was IV in nearly 90%. The subjects underwent highly invasive procedures of total laryngectomy with esophageal reconstruction, lymph node dissection, and preoperative or postoperative chemoradiotherapy. Due to loss of voice, 27.8% lost their employment before surgery, and this rate increased to 40% at 1 year after hospital discharge, suggesting that loss of voice affected occupation status. Some patients developed depression or were downsized. Lymph node dissection simultaneously with total laryngectomy, in which muscles in the cervical and shoulder areas were also resected in wide-ranging surgery, was performed in 90% of the subjects. These patients were discharged from hospital with significantly lower physical functions, in addition to dealing with such effects as a permanent trachea hole, dysphagia, and constipation. Development of respiratory problems after discharge also suggests that patients may have a sense of life crisis and pain caused by inflammation. They may also have a decreased appetite due to dysphagia and reduced food intake may cause insufficient water content. Constipation may develop because patients cannot strain during a bowel movement due to resection of the vocal cord. All these effects damage basic life functions and support for these issues is required for 1 year after discharge following laryngectomy.

### Changes in social adjustment after laryngectomy

The mean SF score of 42 points before surgery significantly decreased to 33.5 points at 3 months after hospital discharge. In addition, as seen in Fig. 1, subjects with an occupation had a score of 29.7 points, which was lower than that of subjects without an occupation. This is a new finding that patients with an occupation have lower social functioning than those without an occupation. This finding suggests that even when subjects with an occupation could go back to work, they might have lost confidence in themselves due to changes in their working environment and might have other difficulties in their daily life, including economic difficulties personally and for their families. Based on the characteristics of patients after laryngectomy, time-dependent changes might have been smaller in subjects without an occupation because these subjects included retired persons, in whom anxiety is limited to their own lives, although they had some feeling of loss. These findings suggest that support is necessary for social adjustment of patients with an occupation.

### Need for support for social adjustment of patients with an occupation

In the main effect test, there was a difference by age in SF, which tended to affect time and diagnosis. This is related to the significant decrease in social functioning of younger patients with an occupation to ≥ 20 to < 40 points at 3 months after hospital discharge and did not improve until 6 months after discharge. SF decreased after laryngectomy in patients with hypopharyngeal cancer, and it is important to provide support for these patients. MH was affected by age and family structure. Patients living alone had higher scores than those who lived with one or more family members, which suggested that MH was better in older patients. SF and MH were significantly higher in older subjects in the third-order interaction, which suggests that support is needed for a return to work or restart of work in younger patients. MH after laryngectomy was better in patients who lived with one or more family members, indicating the importance of family support. However, such patients have difficulty continuing their work to support their family, and this might explain the decreased scores at 3 months after discharge. The time-dependent changes and interactions of social adjustment in patients with and without an occupation at each age (Fig. 2) show that SF and MH were generally lower in younger subjects, which is an important problem.

In subjects aged 34–66 years, Isaksson et al. [19] found that those with an occupation had higher QOL (emotional and social function) than those without an occupation, and this matches the results for younger subjects in our study. However, there are several differences between the study of Isaksson et al. and the current study: follow-up observation was performed at diagnosis, and at 6 months and 1 and 2 years after discharge in Isaksson et al., while we performed follow-up observation before surgery, and at 3 and 6 months and 1 year after discharge. The decrease of SF and MH that we observed at 3 months after discharge was a particularly important finding.

A focus should be placed on the higher scores in older patients, and the lower MH in younger patients who lived with their family compared to those who lived alone. This might be because these subjects were worrying about the lives of their family members and economic problems. Almost 30% of subjects who had to leave work before surgery did so due to loss of voice, and some faced restructuring or developed depression after hospital discharge. This suggests that younger subjects had a problem of social adjustment in the year after discharge. Especially, since subjects with an occupation had higher SF and MH after discharge, compared to those without an occupation, their social role was important to support their life. Therefore, families and colleagues need to understand concrete ways to communicate with younger patients after hospital discharge, so that such patients can maintain or obtain social roles. For example, a patient should have a meeting with a superior at work before or after discharge, if the patient agrees with this idea.

In a previous comparison of patients with partial and total resection of the larynx, those with total laryngectomy showed lower levels of physical function, role functioning (physical), entire sense of well-being, vitality, social functioning, and role functioning (mental) [20]. A comparison of patients with total laryngectomy and US national standards also showed that these patients had significantly lower physical function and role functioning (physical) [14]. Scores for these factors were also low in our study, which indicates that patients with resection of the larynx have to live with significantly lowered physical function after hospital discharge. Since such patients have significant difficulty in daily life for 1 year after discharge, it is important to promote their social adjustment while providing them with physical support.

### Production of a voice close to the real voice

Totally laryngectomized patients have lower QOL for physical function, role functioning, social functioning, general QOL, and pain, compared to patients who received insertion of a prosthesis in total laryngectomy or those with larynx preservation [21]. Almost 30% of our subjects had to leave their work due to loss of voice, and it is important for such patients to obtain a voice close to their real voice for social adjustment. Patients with an occupation began to show lower social functioning 3 months after discharge, compared to those without an occupation, which suggests a sense of loss of their social roles due to loss of their voice. In addition, subjects living with their family had poorer mental health than those living alone, which may have been due to poor communication with family members, resulting in the development of depression. In addition, as reported previously, such patients have a risk of becoming reclusive with no conversations with their family [6].

The incidence of laryngeal and hypopharyngeal cancer is 7.5% [22], which is lower than that for many other types of cancer and, thus, fewer strategies for management of laryngeal and hypopharyngeal cancer have been developed. However, patients with cancer experience a major loss of self when they lose their voice. Iritani [23] suggested that production of voice is an important function that expresses personal characteristics, and some people consider that loss of voice may be the worst event in life [24]. Laryngeal and hypopharyngeal cancer develops in elderly people who are often close to retirement and this may be reflected in the reasons for leaving work. Some of the patients with an occupation faced restructuring due to difficulty in being understood by their colleagues. There are fewer problems if such patients can work without use of their voice, but this is only possible in a few workplaces. If patients cannot communicate with their colleagues smoothly, problems caused by communication variance may occur. A small misunderstanding can accumulate and such patients may be evaluated negatively, leading to restructuring of their work position. The disease is not understood well in most workplaces, and there is a need to promote this understanding among colleagues of patients.

## Conclusion

Regarding the main effect on SF, only age was significant, and SF of subjects aged ≥ 64 years old was favorable. Regarding MH, age and family structure were significant, and older subjects who lived alone had better MH. There was no second-order interaction between time and the other four variables, but there were significant third-order interactions for time/age/occupation for SF and MH. The interaction of time with the three other variables was not significant. Regarding MH, only time/age/occupation was significant. For SF, a weak interaction was suggested, but it was not significant. During the period from 3 to 6 months after discharge, scores decreased more in subjects with an occupation than in those without an occupation, suggesting development of a problem of social adjustment. A public information program may be required to ensure that families and co-workers understand the disease and assist in maintenance or development of social roles of younger patients.

### Limitations

The limitations of the study include the small number of subjects, which eventually reached 27 because long-term participation was required from before surgery to 1 year after discharge. A problem with multiple testing is possible due to this sample size. Surgical method, lymph node dissection, chemoradiotherapy, and disease stage should also have been examined as causal factors, but diagnosis alone was used because of the biased distribution for the other variable and the small number of subjects. We used answers from patients with relatively stable mental and physical conditions because we aimed to obtain information about their actual situations. A future task will be to improve understanding of laryngeal and hypopharyngeal cancer among family members and colleagues of patients. We hope to study the effects of a support system that allows patients with this disease to live as normal a life as possible after hospital discharge.
